# A survey of tube spiders (Araneae, Anyphaenidae) from Jinggangshan National Nature Reserve, Jiangxi Province, China

**DOI:** 10.3897/BDJ.12.e133875

**Published:** 2024-09-13

**Authors:** Zhongjing Wang, Ziying Tang, Yanbin Yao, Shihui Huang, Keke Liu

**Affiliations:** 1 College of Life Science, Jinggangshan University, Ji'an, China College of Life Science, Jinggangshan University Ji'an China; 2 Jinshan College of Fujian Agriculture and Forestry University, Fuzhou, China Jinshan College of Fujian Agriculture and Forestry University Fuzhou China

**Keywords:** Tube spider, survey, taxonomy, distribution

## Abstract

**Background:**

Although there have been many recent taxonomic revisions and large-scale faunistic surveys focusing on spiders from Jiangxi Province, many taxa still remain unknown, such as the Anyphaenidae spiders. Therefore, none of the anyphaenid species has been recorded from this Province.

**New information:**

Anyphaenidae spiders were collected from Jinggangshan National Nature Reserve, Jiangxi Province, China, during the past ten years. A new tube spider species, *Anyphaenaleidashi* Yao & Liu, **sp. nov.** is diagnosed, described and illustrated. Moreover, three species including *A.mogan* Song & Chen, 1987, *A.wuyi* Zhang, Zhu & Song, 2005 and *Rathalosxiushanensis* (Song & Zhu, 1991) are recorded from Jiangxi Province for the first time. Distributions records are given for all investigated species.

## Introduction

The spider family Anyphaenidae is one of the largest families of spiders in the world. They usually occur in the foliage of trees, leaf litter, sometimes in bamboo crust. It consists of 646 species in 58 genera (World Spider Catalog 2024). Of these, 15 species in 3 genera have been reported from China, including *Anyphaenacibagou* Wang & Mi, 2024 (Xizang), *A.grovyle* Lin & Li, 2021 (Hainan), *A.linzhi* Wang & Mi, 2024 (Xizang), *A.mogan* Song & Chen, 1987 (Zhejiang and Hunan), *A.rhynchophysa* Feng, Ma & Yang, 2012 (Yunnan), *A.sceptile* Lin & Li, 2021 (Hainan), *A.shenzhen* Lin & Li, 2021 (Guangdong), *A.shufui* Wang & Mi, 2024 (Xizang), *A.tibet* Lin & Li, 2021 (Xizang), *A.wuyi* Zhang, Zhu & Song, 2005 (Fujian, Guizhou and Taiwan), *A.yejiei* Wang & Mi, 2024 (Xizang), *A.taiwanensis* Chen & Huang, 2011 (Taiwan), *Rathalostreecko* (Lin & Li 2021) (Hainan), *R.xiushanensis* (Song & Zhu, 1991) (Sichuan, Hubei and Chongqing), *Sinophaenabivalva* (Zhang & Song, 2004) (Guangxi) and *S.xiweni* Lin & Li, 2021 (Sichuan) (World Spider Catalog 2024). It seems these species are distributed widely in the south area of this huge country. However, no species were recorded from Jiangxi Province.

Jiangxi Province is not a biodiversity hotspot for researchers and has not been given enough attention in last ten years in China. Recently, about 100 new species have been discovered from Jiangxi Province, including those such as Agelenidae (Liu et al. 2020a, Liu et al. 2021), Dictynidae (Liu et al. 2018), Oonopidae (Liu et al. 2016, Liu et al. 2019), Phrurolithidae (Liu et al. 2020b, Liu et al. 2020c, Liu et al. 2021, Liu et al. 2022d, Liu et al. 2023), Salticidae (Liu et al. 2017b, Ying et al. 2021, Liu et al. 2022a), Thomisidae (Liu et al. 2017a, Liu et al. 2022c), Gnaphosidae (Liu et al. 2022b), Leptonetidae (Liu et al. 2023) and Anyphaenidae (this study). These discoveries in species diversity are well demonstrated by spiders. However, there are still many species requiring study. While examining spiders collected from Jinggangshan National Nature Reserve in southwest area of Jiangxi, we found these four species belonging to *Anyphaena*, described a new species with photographs and provided distribution map for them.

## Materials and methods

Specimens were examined using a SZ6100 stereomicroscope. Both male and female copulatory organs were dissected and examined in 80% ethanol using an Olympus CX43 compound microscope with a KUY NICE CCD camera. Epigynes were cleared with pancreatin solution (Álvarez-Padilla and Hormiga 2007). Maps were made using the software Arcgis (V. 10.8). Specimens, including dissected male palps and epigynes, were preserved in 75% ethanol after examination. Types are deposited in the Animal Specimen Museum, College of Life Science, Jinggangshan University (ASM-JGSU).

The measurements were taken using a stereomicroscope (AxioVision SE64 Rel. 4.8.3) and are given in millimeters. The body lengths of all specimens exclude the chelicerae and spinnerets. Terminology of the male and female genitalia follows Ramírez (2014) and Baba and Tanikawa (2017). The abbreviations used in the figures and text are: ALE − anterior lateral eye, AME − anterior median eye, At – atrium, CD − copulatory duct, CO − copulatory opening, Con – conductor, d – dorsal, Em – embolus, FD − fertilization duct, Fe – femur, GA − glandular appendage, MA − median apophysis, p – prolateral, Pa – patella, r – retrolateral, RTA − retrolateral tibial apophysis, Spe – spermatheca, Ti – tibia, v – ventral.

## Taxon treatments

### Anyphaena leidashi

Yao & Liu sp. nov.

113B6CD4-E0BE-514B-B175-64283D216003

#### Materials

- **Type status:** Holotype. **Occurrence:** recordedBy: Liu Ke-Ke; individualCount: 1; sex: male; lifeStage: adult; occurrenceID: 15139ABE-9547-5FEF-8F47-A550BF4F667B; **Taxon:** scientificName: *Anyphaenaleidashi* Yao & Liu, sp. nov.; **Location:** country: China; stateProvince: Jiangxi; locality: Ji’an City, Jinggangshan County Level City, Jingzhushan; verbatimElevation: 1105 m; verbatimCoordinates: 26°32'42.39"N, 114°06'35.15"E; georeferenceProtocol: GPS; **Event:** samplingProtocol: handing; eventDate: 02-23-2024
- **Type status:** Paratype. **Occurrence:** recordedBy: Liu Ke-Ke; individualCount: 1; sex: male; lifeStage: adult; occurrenceID: 6D8DC4E3-3A72-5037-BC18-E32BA7DDAB03; **Taxon:** scientificName: *Anyphaenaleidashi* Yao & Liu, sp. nov.; **Location:** country: China; stateProvince: Jiangxi; locality: Ji’an City, Jinggangshan County Level City, Jingzhushan; verbatimElevation: 1105 m; verbatimCoordinates: 26°32'42.39"N, 114°06'35.15"E; georeferenceProtocol: GPS; **Event:** samplingProtocol: handing; eventDate: 02-23-2024
- **Type status:** Paratype. **Occurrence:** recordedBy: Liu Ke-Ke; individualCount: 1; sex: female; lifeStage: adult; occurrenceID: 478D3C51-DFFB-57B5-B484-2459577D40F8; **Taxon:** scientificName: *Anyphaenaleidashi* Yao & Liu, sp. nov.; **Location:** country: China; stateProvince: Jiangxi; locality: Ji’an City, Jinggangshan County Level City, Jingzhushan; verbatimElevation: 1105 m; verbatimCoordinates: 26°32'42.39"N, 114°06'35.15"E; georeferenceProtocol: GPS; **Event:** samplingProtocol: handing; eventDate: 02-23-2024
- **Type status:** Paratype. **Occurrence:** recordedBy: Liu Ke-Ke; individualCount: 1; sex: female; lifeStage: adult; occurrenceID: B515DA92-77E9-5F48-BB11-129270911178; **Taxon:** scientificName: *Anyphaenaleidashi* Yao & Liu, sp. nov.; **Location:** country: China; stateProvince: Jiangxi; locality: Ji’an City, Jinggangshan County Level City, Jingzhushan; verbatimElevation: 1105 m; verbatimCoordinates: 26°32'42.39"N, 114°06'35.15"E; georeferenceProtocol: GPS; **Event:** samplingProtocol: handing; eventDate: 02-23-2024
- **Type status:** Paratype. **Occurrence:** recordedBy: Liu Ke-Ke; individualCount: 1; sex: female; lifeStage: adult; occurrenceID: 292F495E-2BD3-580C-A324-11C246288F65; **Taxon:** scientificName: *Anyphaenaleidashi* Yao & Liu, sp. nov.; **Location:** country: China; stateProvince: Jiangxi; locality: Ji’an City, Jinggangshan County Level City, Jingzhushan; verbatimElevation: 1105 m; verbatimCoordinates: 26°32'42.39"N, 114°06'35.15"E; georeferenceProtocol: GPS; **Event:** samplingProtocol: handing; eventDate: 02-23-2024
- **Type status:** Paratype. **Occurrence:** recordedBy: Liu Ke-Ke; individualCount: 1; sex: female; lifeStage: adult; occurrenceID: B3132E8F-050F-51BB-8497-07F62FF79334; **Taxon:** scientificName: *Anyphaenaleidashi* Yao & Liu, sp. nov.; **Location:** country: China; stateProvince: Jiangxi; locality: Ji’an City, Jinggangshan County Level City, Jingzhushan; verbatimElevation: 1105 m; verbatimCoordinates: 26°32'42.39"N, 114°06'35.15"E; georeferenceProtocol: GPS; **Event:** samplingProtocol: handing; eventDate: 02-23-2024
- **Type status:** Paratype. **Occurrence:** recordedBy: Liu Ke-Ke; individualCount: 1; sex: female; lifeStage: adult; occurrenceID: 32DE1D7C-70A2-525C-A0F1-B640B1B0FC1D; **Taxon:** scientificName: *Anyphaenaleidashi* Yao & Liu, sp. nov.; **Location:** country: China; stateProvince: Jiangxi; locality: Ji’an City, Jinggangshan County Level City, Jingzhushan; verbatimElevation: 1105 m; verbatimCoordinates: 26°32'42.39"N, 114°06'35.15"E; georeferenceProtocol: GPS; **Event:** samplingProtocol: handing; eventDate: 02-23-2024
- **Type status:** Paratype. **Occurrence:** recordedBy: Liu Ke-Ke; individualCount: 1; sex: female; lifeStage: adult; occurrenceID: 599D1805-CEAD-584E-BCB6-7389399AFCBA; **Taxon:** scientificName: *Anyphaenaleidashi* Yao & Liu, sp. nov.; **Location:** country: China; stateProvince: Jiangxi; locality: Ji’an City, Jinggangshan County Level City, Jingzhushan; verbatimElevation: 1105 m; verbatimCoordinates: 26°32'42.39"N, 114°06'35.15"E; georeferenceProtocol: GPS; **Event:** samplingProtocol: handing; eventDate: 02-23-2024
- **Type status:** Paratype. **Occurrence:** recordedBy: Liu Ke-Ke; individualCount: 1; sex: female; lifeStage: adult; occurrenceID: BD6B7ACC-D65C-53D3-A464-11E994522F9D; **Taxon:** scientificName: *Anyphaenaleidashi* Yao & Liu, sp. nov.; **Location:** country: China; stateProvince: Jiangxi; locality: Ji’an City, Jinggangshan County Level City, Jingzhushan; verbatimElevation: 1105 m; verbatimCoordinates: 26°32'42.39"N, 114°06'35.15"E; georeferenceProtocol: GPS; **Event:** samplingProtocol: handing; eventDate: 02-23-2024

#### Description

**Figure 1.**
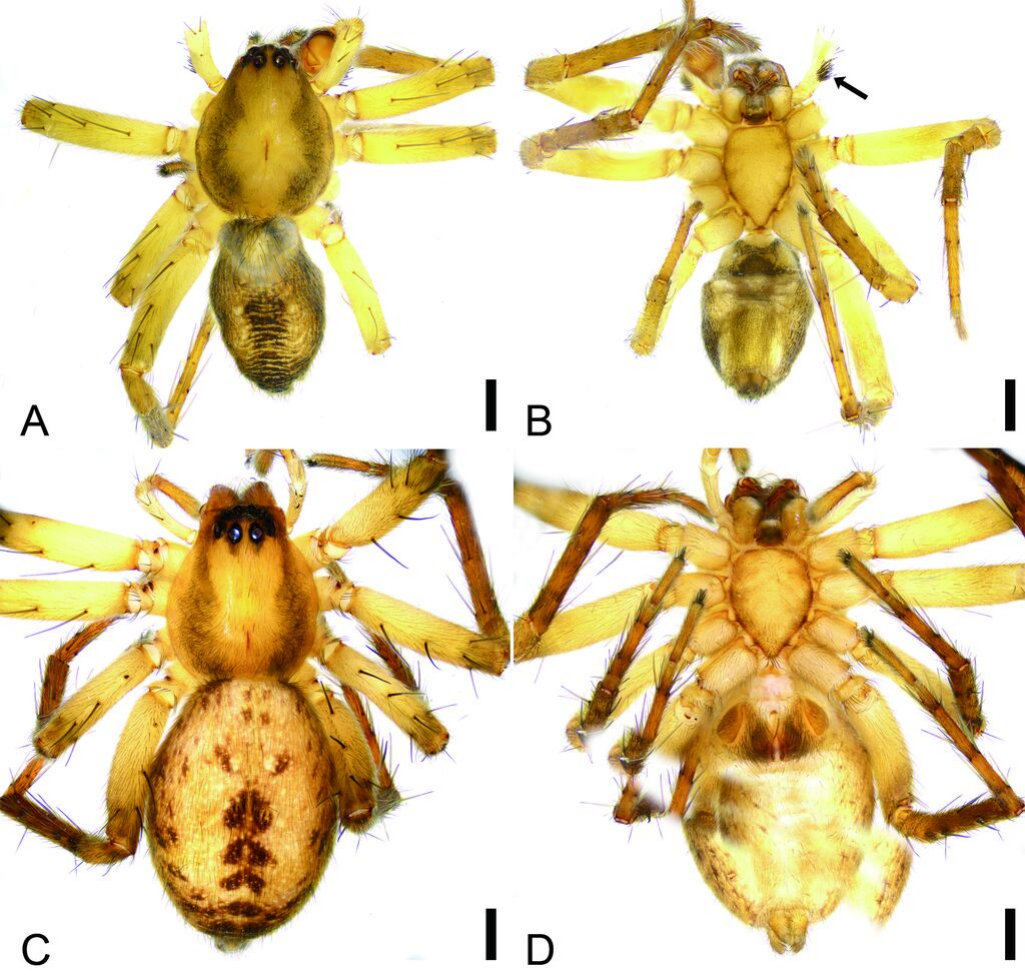
*Anyphaenaleidashi* sp. nov., habitus. **A, B** male holotype, dorsal and ventral views, black arrow shows the palpal femur with a cluster of strong spines proximally; **C, D** female paratype, dorsal and ventral views. Scale bars: 0.5 mm.

**Male** (holotype) (Fig. 1A, B). Total length 3.43 mm.

Carapace 1.72 long, 1.43 wide. Eye sizes and interdistances (Fig. 1A): AME 0.07, ALE 0.11, PME 0.11, PLE 0.12, AME−AME 0.05, AME−ALE 0.03, PME−PME 0.13, PME−PLE 0.1, AME−PME 0.18, AME−PLE 0.21, ALE−ALE 0.26, PLE−PLE 0.49, ALE−PLE 0.07. MOA 0.33 long, front width 0.19, back width 0.3. Chelicerae (Fig. 1B) with a long fang and three promarginal and five retromarginal teeth. Endites (Fig. 1B) longer than wide. Labium wider than long. Sternum (Fig. 1B) oval, anteriorly flat, posterior end blunt. Legs (Fig. 1A, B): measurements: I 4.43 (1.22, 0.51, 1.09, 0.96, 0.65); II 5.35 (1.51, 0.7, 1.43, 1.1, 0.61); III 4.43 (1.23, 0.57, 1.05, 1.06, 0.52); IV 5.89 (1.62, 0.71, 1.38, 1.57, 0.61); spination: I Fe: d4; Ti: p4, r2, v6; Mt: d4, p3, r2, v2; II Fe: d2; Ti: d1, p3, r3, v5; Mt: d4, p2, r3, v2; III Fe: d4 ; Ti: d2, p2, r2, v4; Mt: d6, p4, r3, v4; IV Fe: d4; Ti: d3, p4, r3, v3; Mt: d6, p5, r4, v6. Abdomen (Fig. 1A, B) 1.57 long, 1.25 wide.

**Coloration** (Fig. 1A, B). Carapace yellow to brown, medially yellow, laterally brown. Chelicerae brown. Endites yellow. Labium brown. Sternum yellow, with brown lateral margin. Legs yellow to pale brown, femora yellow, patellae, tibiae and metatarsi brown. Abdomen pale brown to dark brown, medially with a broad dark stripe; venter with a broad brown stripe.

**Figure 2.**
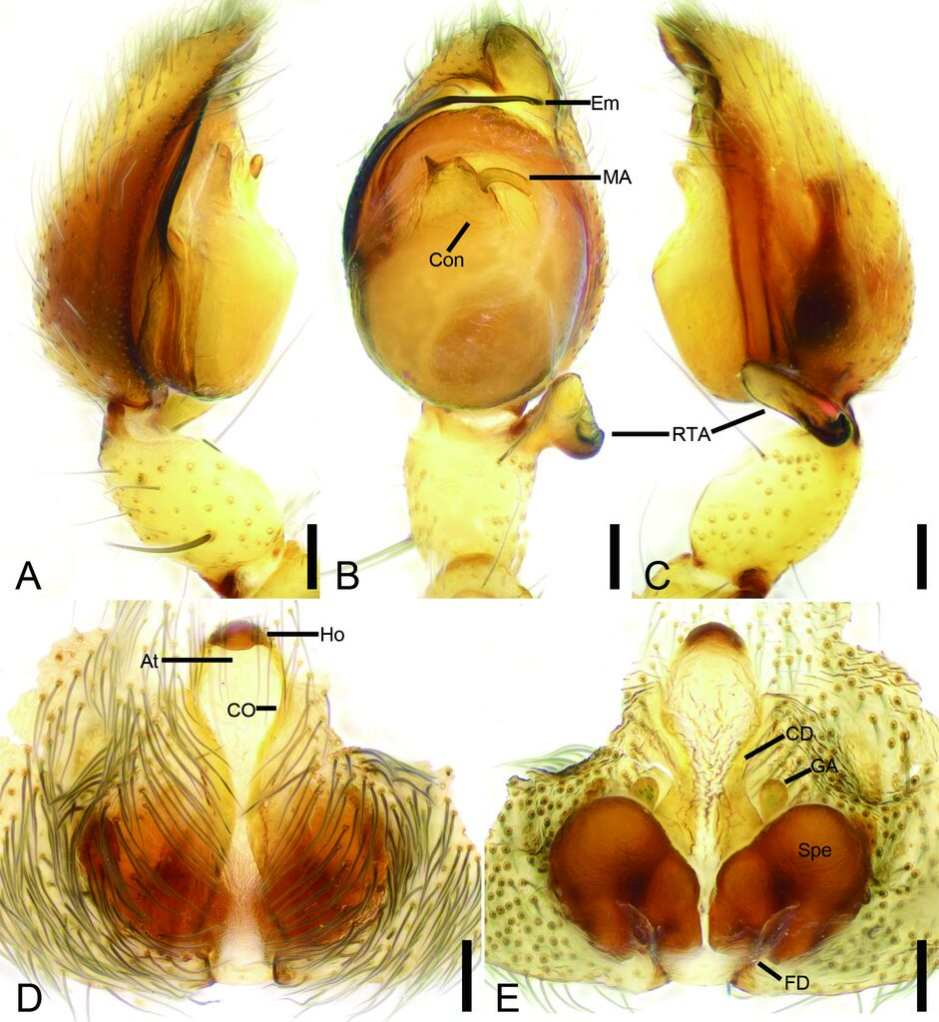
*Anyphaenaleidashi* sp. nov.. **A** palp of holotype, prolateral view; **B** same, ventral view; **C** same, retrolateral view; **D** epigyne of paratype, ventral view; **E** same, dorsal view. Abbreviations: At − atrium; CD − copulatory ducts; CO − copulatory openings; Con − conductor; Em − embolus; FD − fertilization duct; GA − glandular appendages; MA − median apophysis; RTA − retrolateral tibial apophysis; Spe − spermathecae. Scale bars: 0.1 mm.

**Palp** (Fig. 1B and Fig. 2A−C). Femur bears a cluster of strong spines proximally. Retrolateral tibial apophysis strong, shoe-shaped in ventral view, rod-like with an upward thick hook-shaped branch at base in retrolateral view. Median apophysis hook-shaped, curved ventrally. Conductor translucent, trapezoidal, with a triangular-shaped tip. Embolus flagelliform, arising from 9 o’clock and ending at ~ 1 o’clock on tegulum.

**Female.** As in male, except as noted.

**Habitus** as in Fig. 1C, D. As in male, except as noted. Total length 4.48.

Carapace 1.93 long, 1.57 wide. Eye sizes and interdistances (Fig. 1C): AME 0.09, ALE 0.13, PME 0.11, PLE 0.13, AME−AME 0.06, AME−ALE 0.02, PME−PME 0.13, PME−PLE 0.11, AME−PME 0.2, AME−PLE 0.24, ALE−ALE 0.28, PLE−PLE 0.55, ALE−PLE 0.07. MOA 0.38 long, front width 0.23, back width 0.35. Abdomen (Fig. 1C, D) 2.58 long, 1.89 wide. Leg (Fig. 1C, D) measurements: I 6.41 (1.76, 0.8, 1.71, 1.36, 0.78); II 6 (1.69, 0.71, 1.58, 1.27, 0.75); III 4.8 (1.35, 0.64, 1.07, 1.2, 0.54); IV 6.63 (1.91, 0.79, 1.56, 1.68, 0.69); spination: I Fe: d4, p1; Ti: d2, p2, r3, v6; Mt: d4, p2, r2, v2; II Fe: d4, r1; Ti: d2, p2, r2, v2; Mt: d4, p2, r2, v2; III Fe: d4; Pa: r1; Ti: d4, p2, r2, v2; Mt: d6, p2, r4, v4; IV Fe: d4; Pa: r1; Ti: d4, p3, r2, v3; Mt: d6, p4, r2, v6.

**Coloration** (Fig. 1C, D). Lighter than male. Abdomen, with short chevron-shaped dark brown stripes medially, laterally with many dark brown spots.

**Epigyne** (Fig. 2D, E). Epigynal plate longer than wide, antero-medially with a sclerotized hood. Atrium slender, with a large biconvex scutum in medial part of the epigynum. Copulatory openings small, covered by the biconvex scutum. Glandular appendages small, balloon-like, located at the posterior part of copulatory ducts. Copulatory ducts relatively broad, extending from anterior to sub-posterior vulva. Spermathecae oval, large, convergent, with parallel inner margin and a distinct constriction. Fertilization ducts relatively long, located postero-medially, directed anteriorly.

#### Diagnosis

Males of this new species are similar to those of *Anyphaenataiwanensis* Chen & Huang, 2011 (Chen and Huang (2011): 80, figs 4, 5) and *A.yoshitakei* Baba & Tanikawa, 2017 (Baba and Tanikawa (2017): 31, fig. 1F, G) in having the shoe-shaped tibial apophysis with an upward thick hook-shaped branch at base, the hook-shaped median apophysis curved ventrally and the flagelliform embolus, but can be distinguished from it by the translucent trapezoidal conductor with a triangular-shaped tip (vs the oval conductor with a long nose-shaped tip in *A.taiwanensis* and the sub-trapezoidal conductor with a blunt saddle-like tip in *A.yoshitakei*) (Fig. 2A−C). Female of this species can be easily distinguished from that of *A.taiwanensis* (Chen and Huang (2011): 80, figs 8, 9) and *A.yoshitakei* (Baba and Tanikawa (2017): 31, fig. 1C, D) by the slender atrium (vs relatively broad in *A.taiwanensis* and *A.yoshitakei*) and the parallel inner margin of spermathecae (vs slightly splayed in *A.taiwanensis* and convergent in *A.yoshitakei*) (Fig. 2D, E).

#### Etymology

The specific name is a noun in apposition and refers to the type locality, Chinese characters "雷打石".

#### Distribution

**Figure 3.**
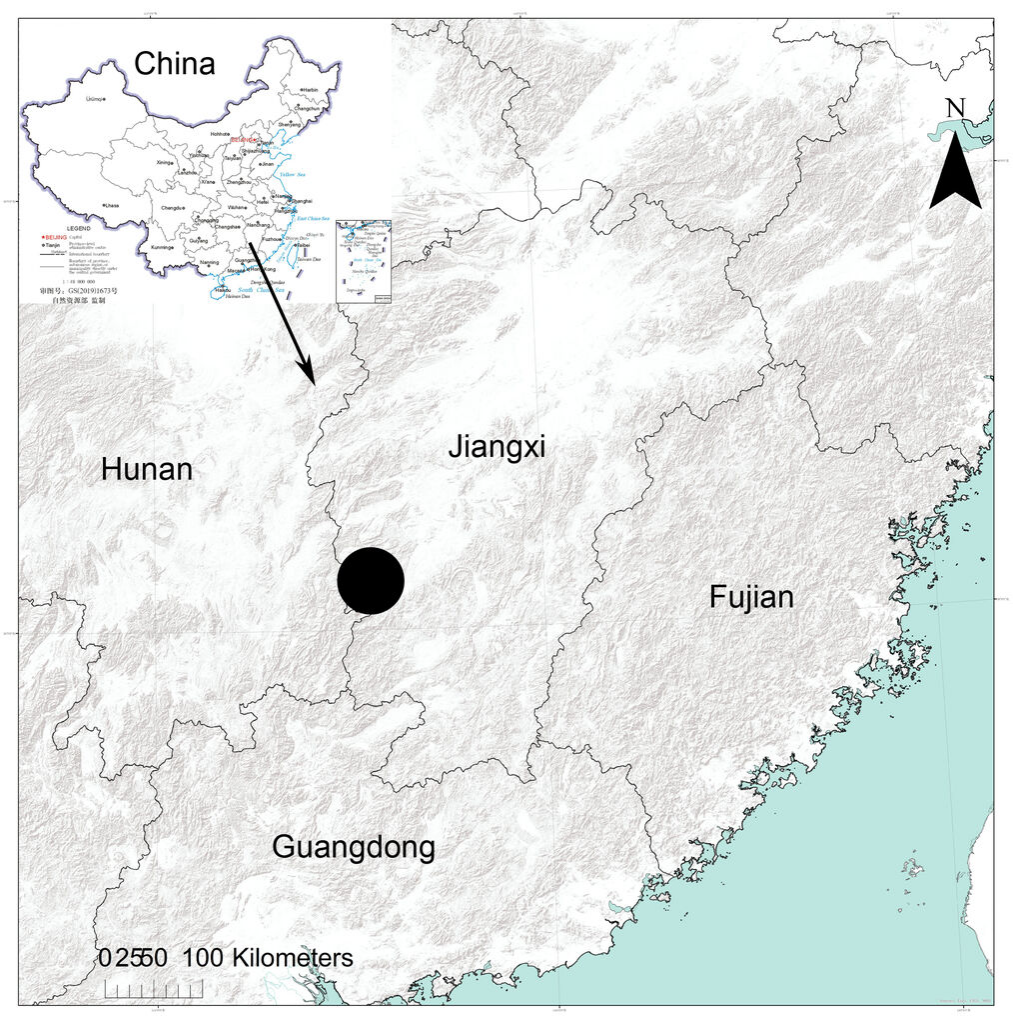
The location of the Jinggangshan National Nature Reserve in China indicated by a large black dot.

Known only from the type locality in Jiangxi Province, China (Fig. 3).

#### Biology

Specimens were mainly collected under the bark in a broad-leaved forest.

### Anyphaena mogan

Song & Chen, 1987

4D56AC81-DEE3-5523-B3FA-6CE8BC3B129F

*Anyphaenamogan* Song & Chen, 1987 - Song and Chen (1987): 13, figs 1−5 (holotype female, not examined; Zhejiang, Huzhou); Chen and Zhang (1991): 260, fig. 273.1-6 (♂♀); Song et al. (1999): 402, fig. 237J−K, N−P (♂♀); Yin et al. (2012): 1064, fig. 557 (♂♀).

#### Materials

- **Type status:** Other material. **Occurrence:** recordedBy: Liu Ke-Ke; individualCount: 1; sex: male; lifeStage: adult; occurrenceID: 2901CB49-F3FA-5E51-8AD4-2CAE45AA6625; **Taxon:** scientificName: *Anyphaenamogan* Song & Chen, 1987; **Location:** country: China; stateProvince: Jiangxi; locality: Ji’an City, Qingyuan District, Dawushan; verbatimElevation: 1031 m; verbatimCoordinates: 26°40'48.69"N, 115°25'07.79"E; georeferenceProtocol: GPS; **Event:** samplingProtocol: handing; eventDate: 10-25-2020
- **Type status:** Other material. **Occurrence:** recordedBy: Liu Ke-Ke; individualCount: 1; sex: male; lifeStage: adult; occurrenceID: 3BDF9393-2041-568A-BFDD-0657DF384603; **Taxon:** scientificName: *Anyphaenamogan* Song & Chen, 1987; **Location:** country: China; stateProvince: Jiangxi; locality: Ji’an City, Jinggangshan County Level City, Jinggangshan National Nature Reserve, Bijiashan Scenic Spot; verbatimElevation: 383 m; verbatimCoordinates: 26°30'55.45"N, 114°12'12.05"E; georeferenceProtocol: GPS; **Event:** samplingProtocol: handing; eventDate: 05-01-2021
- **Type status:** Other material. **Occurrence:** recordedBy: Liu Ke-Ke; individualCount: 1; sex: female; lifeStage: adult; occurrenceID: 5ACDE752-5BA6-52CA-839C-30FB74136CFE; **Taxon:** scientificName: *Anyphaenamogan* Song & Chen, 1987; **Location:** country: China; stateProvince: Jiangxi; locality: Ji’an City, Jinggangshan County Level City, Longtan Scenic Spot; verbatimElevation: 927 m; verbatimCoordinates: 26°31'51.6"N, 114°7'55.2"E; georeferenceProtocol: GPS; **Event:** samplingProtocol: handing; eventDate: 06-01-2014
- **Type status:** Other material. **Occurrence:** recordedBy: Liu Ke-Ke; individualCount: 1; sex: female; lifeStage: adult; occurrenceID: ABF27176-E584-54F1-B9DD-DB58D685558C; **Taxon:** scientificName: *Anyphaenamogan* Song & Chen, 1987; **Location:** country: China; stateProvince: Jiangxi; locality: Ji’an City, Jinggangshan County Level City, Huangao Township, Xiaoxi Forest Farm; verbatimElevation: 365 m; verbatimCoordinates: 26°28'8.4"N, 114°12'36"E; georeferenceProtocol: GPS; **Event:** samplingProtocol: handing; eventDate: 05-30-2017
- **Type status:** Other material. **Occurrence:** recordedBy: Liu Ke-Ke; individualCount: 1; sex: female; lifeStage: adult; occurrenceID: 91310683-DF2B-5DAD-A32A-E2A14E6F92DA; **Taxon:** scientificName: *Anyphaenamogan* Song & Chen, 1987; **Location:** country: China; stateProvince: Jiangxi; locality: Ji’an City, Jinggangshan County Level City, Ciping Town; verbatimElevation: 950 m; verbatimCoordinates: 26°34'12.89"N, 114°07'41.87"E; georeferenceProtocol: GPS; **Event:** samplingProtocol: handing; eventDate: 09-30-2018

#### Description

See Song and Chen (1987) for both sexes.

#### Distribution

Known from Jiangxi (new record, Fig. 3), Hunan (Yin et al. 2012) and Zhejiang (Song and Chen 1987), China.

#### Notes

This species has been reported from Jiangxi Province first time andmay be more widespread in outh China than we actually know.

### Anyphaena wuyi

Zhang, Zhu & Song, 2005

877CF4C2-015C-5C6A-90C2-0D6210B6EBDD

*Anyphaenawuyi* Zhang, Zhu & Song, 2005 - *Zhang et al. (2005)*: 2, figs 1−10 (holotype male, not examined; Fujian, Wuyishan); Chen (2010): 70, figs 1−6 (♂♀); Chen (2012): 36, fig. 10A−E, plate 3C−D (♂♀).

#### Materials

- **Type status:** Other material. **Occurrence:** recordedBy: Liu Ke-Ke; individualCount: 5; sex: male; lifeStage: adult; occurrenceID: 58DC1BBF-F78B-56E9-90AB-D1258B7599BA; **Taxon:** scientificName: *Anyphaenawuyi* Zhang, Zhu & Song, 2005; **Location:** country: China; stateProvince: Jiangxi; locality: Ji’an City, Jinggangshan County Level City, Xiangzhou; verbatimElevation: 459 m; verbatimCoordinates: 26°37'19.2"N, 114°15'54"E; georeferenceProtocol: GPS; **Event:** samplingProtocol: handing; eventDate: 08-06-2015
- **Type status:** Other material. **Occurrence:** recordedBy: Liu Ke-Ke; individualCount: 6; sex: female; lifeStage: adult; occurrenceID: 5D48D8F2-7AAD-520D-ACF1-3A71928CF243; **Taxon:** scientificName: *Anyphaenawuyi* Zhang, Zhu & Song, 2005; **Location:** country: China; stateProvince: Jiangxi; locality: Ji’an City, Jinggangshan County Level City, Xiangzhou; verbatimElevation: 459 m; verbatimCoordinates: 26°37'19.2"N, 114°15'54"E; georeferenceProtocol: GPS; **Event:** samplingProtocol: handing; eventDate: 08-06-2015
- **Type status:** Other material. **Occurrence:** recordedBy: Liu Ke-Ke; individualCount: 1; sex: male; lifeStage: adult; occurrenceID: 04D12BE5-84D5-53B7-9564-C134C86221DC; **Taxon:** scientificName: *Anyphaenawuyi* Zhang, Zhu & Song, 2005; **Location:** country: China; stateProvince: Jiangxi; locality: Ji’an City, Jinggangshan County Level City, Jingzhushan; verbatimElevation: 1146 m; verbatimCoordinates: 26°29'45.6"N, 114°4'44.4"E; georeferenceProtocol: GPS; **Event:** samplingProtocol: handing; eventDate: 12-20-2015
- **Type status:** Other material. **Occurrence:** recordedBy: Liu Ke-Ke; individualCount: 1; sex: female; lifeStage: adult; occurrenceID: E07F35CF-2855-5A7F-9A1F-6D996423627E; **Taxon:** scientificName: *Anyphaenawuyi* Zhang, Zhu & Song, 2005; **Location:** country: China; stateProvince: Jiangxi; locality: Ji’an City, Jinggangshan County Level City,Longtan Scenic Spot; verbatimElevation: 856 m; verbatimCoordinates: 26°35'45.6"N, 114°8'20.4"E; georeferenceProtocol: GPS; **Event:** samplingProtocol: handing; eventDate: 05-31-2014
- **Type status:** Other material. **Occurrence:** recordedBy: Liu Ke-Ke; individualCount: 4; sex: male; lifeStage: adult; occurrenceID: CC0E5AFF-075D-5962-9127-53A6CB6221F8; **Taxon:** scientificName: *Anyphaenawuyi* Zhang, Zhu & Song, 2005; **Location:** country: China; stateProvince: Jiangxi; locality: Ji’an City, Jinggangshan County Level City, Jingzhushan; verbatimElevation: 1105 m; verbatimCoordinates: 26°32'42.39"N, 114°06'35.15"E; georeferenceProtocol: GPS; **Event:** samplingProtocol: handing; eventDate: 02-23-2024
- **Type status:** Other material. **Occurrence:** recordedBy: Liu Ke-Ke; individualCount: 1; sex: female; lifeStage: adult; occurrenceID: 7C27D370-FE56-5666-B784-2AAD3123B7D8; **Taxon:** scientificName: *Anyphaenawuyi* Zhang, Zhu & Song, 2005; **Location:** country: China; stateProvince: Jiangxi; locality: Ji’an City, Jinggangshan County Level City, Jingzhushan; verbatimElevation: 1105 m; verbatimCoordinates: 26°32'42.39"N, 114°06'35.15"E; georeferenceProtocol: GPS; **Event:** samplingProtocol: handing; eventDate: 02-23-2024

#### Description

See Zhang et al. (2005) for both sexes.

#### Distribution

Known from Jiangxi (new record, Fig. 3), Fujian (Zhang et al. 2005), Guizhou (Zhang et al. 2005) and Taiwan (Chen 2010), China.

#### Notes

This species has been reported from Jiangxi Province first time. The distribution may be more widespread in South China than we actually know. This hypothesis will be confirmed or rejected in the future when more materials can be collected.

### Rathalos xiushanensis

(Song & Zhu, 1991)

91D49724-6D48-520C-8689-96E5782FE031

*Anyphaenaxiushanensis* Song & Zhu, 1991- Song and Zhu (1991): 1, figs 1−5 (holotype female, not examined; Chongqing, Xiushan Tujia and Miao Autonomous County); Song and Li (1997): 417, fig. 21A−E (♂♀); Song et al. (1999): 402, fig. 237L−M, Q−R (♂♀).
*Rathalosxiushanensis* Lin et al. 2022: 201, fig. 1A−E (transferred from *Anyphaena*).

#### Materials

- **Type status:** Other material. **Occurrence:** recordedBy: Liu Ke-Ke; individualCount: 1; sex: male; lifeStage: adult; occurrenceID: CEAA7778-FD5B-5016-9D39-5D379590775D; **Taxon:** scientificName: *Rathalosxiushanensis* (Song & Zhu, 1991); **Location:** country: China; stateProvince: Jiangxi; locality: Ji’an City, Jinggangshan County Level City, Xiangzhou Township,Baishuizhai; verbatimElevation: 375 m; verbatimCoordinates: 26°36'10.8"N, 114°15'28.8"E; georeferenceProtocol: GPS; **Event:** samplingProtocol: handing; eventDate: 05-29-17
- **Type status:** Other material. **Occurrence:** recordedBy: Liu Ke-Ke; individualCount: 1; sex: male; lifeStage: adult; occurrenceID: C2C2D3CD-28C9-5E79-81B9-9BD586C0F5D2; **Taxon:** scientificName: *Rathalosxiushanensis* (Song & Zhu, 1991); **Location:** country: China; stateProvince: Jiangxi; locality: Ji’an City, Jinggangshan County Level City, Maoping Township, Bajiaolou Scenic Spot; verbatimElevation: 418 m; verbatimCoordinates: 26°28'19.2"N, 114°12'7.2"E; georeferenceProtocol: GPS; **Event:** samplingProtocol: handing; eventDate: 05-30-17

#### Description

See Song and Zhu (1991) for both sexes.

#### Distribution

Known from Jiangxi (new record, Fig. 3), Sichuan (Song and Zhu 1991), Hubei (Song and Zhu 1991, Lin et al. 2022) and Chongqing (Lin et al. 2022), China.

#### Notes

The genus *Rathalos* Lin & Li, 2022 was established by Lin et al. (2022) based on the type species of *Anyphaenaxiushanensis*. Besides the type locality, it has been recorded from bushes in diverse locations of Jiangxi, Sichuan and Hubei. It seems that widely distributed in southern China. This hypothesis will be confirmed or rejected in the future when more materials can be collected.

## Discussion

Occurrence of these four anyphaenida species, *Anyphaenaleidashi* sp. nov., *A.mogan* Song & Chen, 1987, *A.wuyi* Zhang, Zhu & Song, 2005 and *Rathalosxiushanensis* (Song & Zhu 1991) in Jinggangshan National Nature Reserve unexpected one of the National Nature Reserves from Jiangxi rovince. The highest species diversity of nyphaenida in South China was reported in Xizang (five species) and Hainan (four species) (Lin et al. 2021, Lin et al. 2022, Li et al. 2024, World Spider Catalog 2024). diversity on anyphaenids species in Jiangxi is relatively high. It suggests that some anyphaenida species will also be found in other Nature Reserves or mountains in Jiangxi Province. However, this still needs to be confirmed by future collecting works.

## Supplementary Material

XML Treatment for Anyphaena leidashi

XML Treatment for Anyphaena mogan

XML Treatment for Anyphaena wuyi

XML Treatment for Rathalos xiushanensis
